# Comparative Pathology

**Published:** 1894-12

**Authors:** J. G. Adami

**Affiliations:** Professor of Pathology in McGill University


### COMPARATIVE PATHOLOGY.

### REPORTS OF CASES.

---

By J. G. Adami, M.A., M.D.,

Professor of Pathology in McGill University. With Introductory Notes by Wesley Mills, M.D ,D.V.S., Professor of Physiology and Lecturer on Cynology in the Faculty of Comparative Medicine and Veterinary Science in McGill University.

---

[Dr. Andrew Darling has published a report of the case to which the subsequent pathological examination detailed below refers, in the January number of this Journal.]

This gentleman, knowing my interest in all that relates to dogs more especially, handed over the specimens to me, believing them to be rare and valuable. I thought that I could serve the profession and pathology best by putting them into the hands of so competent an expert as Dr. Adami is known to be, and think the report he has made will be an endorsement of my course.

The theory of causation he gives seems to me in every way reasonable, and to be borne out, in a measure, by the history of the case.

Doubtless if regular autopsies were made in all cases, kidney disease among the lower animals, and especially in dogs, might not be found to be so rare as is commonly supposed.

Dr. Adami reports as follows:

In the month of March of this year, I received from Dr. Wesley Mills the kidneys of a dog, presenting to the naked eye the condition of chronic interstitial nephritis. At the time of receiving them I was very fully employed otherwise. Since then I have made examination of the organs by various methods, and the following description of this, which, I believe, is an unusual condition in the dog as in all the other domestic animals, may be of interest.

The two kidneys differ very considerably in size; the right is the larger, measuring 7.5 ctm. in length by 4.2 ctm. in breadth (of actual kidney substance in the middle of the organ, from internal to external surface) [3x1⅝ inches]. Similar measurements of the left kidney give 5.3 by 3.4 ctm., or 2½ and 1¼ inches respectively. The capsules of both are thick, but peeled off with greater ease than had been expected, revealing a coarsely nodular surface. Both organs were firm on section, and presented somewhat dilated pelves, patent and extending longitudinally. The cortex varied greatly in thickness, in parts it appeared even broader than normal, in other alternating regions, corresponding to the depressions seen at the surface, it was almost entirely, if not quite atrophied. On the whole, the cortex of the right kidney was broader and less affected than that of the left. I do not give measurements, inasmuch as the canine kidney varies so greatly in dimensions, that such measurements would have but little meaning.

Portions of both kidneys were hardened in alcohol, cut both by the freezing and paraffin methods, and stained in various ways.

The sections obtained presented the appearance that is so well known in the chronic interstitial nephritis of man, that is to say, in the atrophic kidney of gout and other conditions, accompanied by arterio-sclerosis. There was found a chronic thickening of the arterial walls, with much fibrous development around the arteries leading to the cortex.

The cortical region itself presented shrunken, contracted areas, characterized by extreme development of fibrous tissue, and consequent atrophy of the tubules, alternating with areas, in which the tubules had become greatly distended, in fact almost cystic. There was a general fibrosis of the medulla of the kidney. Some of the tubules contained a small amount of uratic deposit, and in the pelvis of the left kidney, at the lower extremity, was a minute calculus. Both ureters had rather thickened walls.

In certain respects the appearances differed from what is usually seen in man. Thus, the distended tubules retained a well defined, single layer of flattened cells of renal epithelium; the basement membrane beneath this was peculiarly thickened, and, stained with hæmatoxylin, took the deep tint characteristic of calcareous infiltration. So, too, the areas of fibrous infiltration, or overgrowth, were richer in nuclei than is usual in the human gouty kidney. In general, however, the sections might easily be mistaken for those of an advanced condition of interstitial nephritis in man.

The condition of the ureters and pelves was not sufficiently advanced to render it certain that there had been any marked degree of obstruction to the flow of urine, either in the lower portions of the ureters or in the bladder.

That the dog should present this condition of cirrhosis of the kidney, or interstitial nephritis, is not surprising, when it is remembered that in man the condition is so frequently associated with excessive nitrogenous diet, coupled with insufficient exercise, and that the dog is most liable to be subjected to these very influences. I have seen well marked cirrhosis of the kidneys in the pig, another animal resembling man and the dog in these respects.

Like man, the dog is liable to eczematous skin eruptions, associated, very probably, with unhealthy diet; the dog also is, I have found, the frequent subject of fibroid changes in the valves of the heart. I must, however, acknowledge that my opportunities for making necropsies on the dog have not been so frequent that I can recall other cases of vascular disturbance (and this condition of the kidney is closely associated with chronic arterial change). Possibly a slight amount of arterial thickening and interstitial nephritis is comparatively common. It would be interesting to learn the experiences of others in this respect.

The woodchuck "Arctomys Monax," referred to below, was in my possession for about two and one-half years. The specimen was under constant observation, as I was engaged in the study of hibernation in this and other animals. During the greater part of this period it seemed to be in perfect health. It was kept most of the time in my pigeon loft, which was comfortable at all times, and during all but the winter season was freely open. The animal itself was kept in a strong wire cage and fed upon stale bread, carrots, turnips, apples, etc.

As usual, in the spring it was low in flesh, poor in coat, and generally in low vital condition. However, beyond this, which is the condition of all the specimens I have kept in confinement, there was little to be observed. It was found dead in its cage on May 4 of the present year. Upon making an autopsy no signs of organic disease were discovered, except in the liver and in the omentum, into which there was extravasation of blood. Some of this had escaped and clotted just outside the peritoneum, so that hæmorrhage may have been the immediate cause of death. Professor Adami's detailed report follows.

The lobe of liver sent for examination (apparently the right lobe), has, upon its under surface, the distended gall bladder. To the outer side of this is situated a large mass, sharply defined from the rest of the liver tissue, whose length antero-posteriorly is 3.0 ctm., depth (from side to side) 1.8 ctm., and breadth (from below downwards) is 3.3 ctm., in the hardened specimen. This tumor occupies the extremity of the lobe, bulging very markedly on both surfaces of the lobe. It is covered by atrophied liver tissue, which it has displaced. On section the tissue of the tumor is found firm, though not so firm as the normal liver tissue, and paler, with numerous greatly distended vessels and areas of hæmorrhage.

Upon microscopic examination, the centre of the growth is found almost entirely angiomatous, with here and there masses of cells of hepatic tissue type. These, in consequence of the hæmorrhage into the central area, fail to take the stain. Towards the periphery the growth assumes the character of an adenoma, being formed of cells resembling, on the whole, those of the liver, being occasionally the seat of fatty infiltrations, but on the other hand, presenting no lobular arrangement, and being smaller than normals.

Angiomata of the liver are relatively common, in fact, of all the internal organs, the liver is the most frequent seat of these vascular overgrowths. But this association of adenoma and angioma is more unusual, and I would rather not express a definite opinion about it until I have had the opportunity of examining sections prepared by several different methods.

